# Neuronal BDNF Signaling Is Necessary for the Effects of Treadmill Exercise on Synaptic Stripping of Axotomized Motoneurons

**DOI:** 10.1155/2015/392591

**Published:** 2015-03-31

**Authors:** Joey Krakowiak, Caiyue Liu, Chandana Papudesu, P. Jillian Ward, Jennifer C. Wilhelm, Arthur W. English

**Affiliations:** ^1^Department of Cell Biology, Emory University School of Medicine, 615 Michael Street, Room 405P, Atlanta, GA 30322, USA; ^2^Department of Plastic and Reconstructive Surgery, Changzheng Hospital, Second Military Medical University, Shanghai, China; ^3^Department of Psychology, College of Charleston, 66 George Street, Charleston, SC 29424, USA

## Abstract

The withdrawal of synaptic inputs from the somata and proximal dendrites of spinal motoneurons following peripheral nerve injury could contribute to poor functional recovery. Decreased availability of neurotrophins to afferent terminals on axotomized motoneurons has been implicated as one cause of the withdrawal. No reduction in contacts made by synaptic inputs immunoreactive to the vesicular glutamate transporter 1 and glutamic acid decarboxylase 67 is noted on axotomized motoneurons if modest treadmill exercise, which stimulates the production of neurotrophins by spinal motoneurons, is applied after nerve injury. In conditional, neuron-specific brain-derived neurotrophic factor (BDNF) knockout mice, a reduction in synaptic contacts onto motoneurons was noted in intact animals which was similar in magnitude to that observed after nerve transection in wild-type controls. No further reduction in coverage was found if nerves were cut in knockout mice. Two weeks of moderate daily treadmill exercise following nerve injury in these BDNF knockout mice did not affect synaptic inputs onto motoneurons. Treadmill exercise has a profound effect on synaptic inputs to motoneurons after peripheral nerve injury which requires BDNF production by those postsynaptic cells.

## 1. Introduction

Despite the fact that axons in the proximal segments of cut peripheral nerves are capable of regenerating and reinnervating their targets, functional recovery after traumatic peripheral nerve injuries is poor [1–3]. There are three main problems thought to contribute to this poor recovery: axons are slow to regenerate, some regenerating axons are misdirected and reinnervate inappropriate targets, and peripheral axotomy produces changes in the circuitry of neurons in the central nervous system (CNS) [4].

The withdrawal of synaptic inputs from the somata and proximal-most dendrites of motoneurons that follows peripheral nerve transection is one such CNS change. Following peripheral nerve transection, more than half of the synaptic inputs onto motoneurons are withdrawn [5–7]. Both excitatory and inhibitory synapses are withdrawn. Many of these synapses are restored over time, regardless of whether the cut axons in the periphery regenerate and reinnervate their muscle targets [6], but synaptic inputs immunoreactive for vesicular glutamate transporter 1 (VGLUT1), originating mainly from primary afferent neurons [8, 9], continue to be withdrawn for long periods and the intraspinal axonal arbors of these neurons become reduced [6]. The net result is a permanent withdrawal of the terminals of afferent neurons arising primarily from muscle spindles that provide length-dependent feedback to the motoneurons. This permanent synaptic withdrawal is accompanied by a decreased amplitude of stimulus-evoked monosynaptic excitatory postsynaptic potentials (EPSPs) recorded in cat motoneurons [10, 11] and a much smaller restored monosynaptic H reflex in rats [12]. The permanent withdrawal of VGLUT1+ inputs is thought to play an important role in the well-documented functional loss of the stretch reflex in self-reinnervated muscles [13–15].

The cellular mechanism by which synapses are withdrawn from motoneurons following peripheral nerve transection remains incompletely known. Initially, reactive astrocytes and/or microglia, which proliferate and surround axotomized motoneurons, were considered [5, 16–19], and immune system-related molecules such as the major histocompatibility complex (MHC) class I molecules [20] and members of the complement family [21] were implicated. However, based on the results of more recent studies [22], a shift in attention to axotomy-induced changes in the motoneurons has occurred. A decline in production of cell adhesion molecules by axotomized motoneurons is known to precede the actual withdrawal of synapses [19, 20, 23], suggesting that these molecules are part of an active, retrograde signaling mechanism that promotes synapse retention. Synaptic withdrawal is found on brainstem motoneurons following transection of the facial or hypoglossal nerves, which do not contain sensory axons ending in VGLUT1+ synaptic terminals [24, 25], consistent with the view that injury-induced synaptic stripping results from a change in the properties of the postsynaptic motoneurons.

Although the immediate withdrawal of most synapses could be explained by a change in retrograde stabilizing signals from the axotomized motoneurons, Alvarez et al. [6] argue persuasively that the permanent withdrawal of VGLUT1+ synaptic terminals from motoneurons following peripheral nerve transection is explained by the effect of damage to the peripheral processes of sensory axons. This notion is also supported by the observation that group I EPSP amplitude in* both* medial and lateral gastrocnemius motoneurons, which each receive monosynaptic inputs from afferent axons in the medial gastrocnemius nerve, is reduced by section of* only* the medial gastrocnemius muscle nerve [10].

One model of a postsynaptic mechanism of synaptic withdrawal includes the retrograde signaling molecule nitric oxide (NO) [26]. Following peripheral axotomy, the production of NO in somatic motoneurons and associated reactive astrocytes is increased. Diffused NO acts directly on presynaptic terminals, activating a signaling pathway eventually leading to microtubule disruption and withdrawal, but it also blocks the secretion of brain derived neurotrophic factor (BDNF) from the axotomized motoneurons at synaptic sites [27], which eventually leads to effects on cytoskeletal actin in the afferent neural terminals and mechanical destabilization of synaptic inputs. The marked and prolonged reduction of neurotrophin expression in motoneurons that is induced by axotomy (reviewed in [28]) is proposed to provide an early signal for profound synaptic withdrawal [25]. In support of the dependence of synaptic stability on retrograde neurotrophic support, no evidence of synaptic withdrawal is noted following prolonged exposure of the proximal stump of a cut eye muscle nerve to the retrogradely transported neurotrophins, BDNF and/or neurotrophin-3 (NT-3) [29]. Additionally, Mendell and colleagues [30] show that prolonged application of recombinant human NT-3 to the proximal segment of a cut muscle nerve in cats results in an increase in group I EPSP amplitudes that are greater than found in intact animals.

Endogenous neurotrophin expression can be induced in motoneurons in the intact spinal cord after modest voluntary exercise [31] or treadmill exercise [32]. Such changes in BDNF expression, as well as that of NT-3, could be involved in the facilitation of synaptic plasticity [33]. We [7, 34, 35] and others [36, 37] have demonstrated that moderate daily treadmill exercise produces* both* an enhancement of axon regeneration and a pronounced effect on synaptic inputs to the axotomized motoneurons after peripheral nerve injury. In the periphery, the enhancing effects of exercise are dependent on the production of BDNF by axotomized neurons [34]. The goal of this study was to determine whether the same neuronal BDNF-dependence underlies the effect of treadmill exercise on synaptic withdrawal following peripheral nerve injury.

In order to test the hypothesized requirement for neuronal BDNF in the effect of treadmill exercise, we studied mice in which the gene for BDNF was knocked out conditionally and in a cell-type specific manner. In mice in which the BDNF gene is knocked out conventionally, homozygous null animals die during the first few postnatal weeks [38, 39]. To avoid this neonatal lethality, we used the Cre-lox system to create conditional BDNF knockout mice. To study the effects of eliminating this gene from postsynaptic motoneurons, we bred mice in which tamoxifen-inducible Cre expression is targeted to neurons in mice in which the coding region of the gene is floxed (BDNF^f/f^). We report here that, if treadmill exercise is applied following peripheral nerve transection, synaptic inputs are withdrawn from motoneurons in which the gene for BDNF was knocked out but not in motoneurons in which the BDNF gene is expressed. Preliminary reports of some of our observations have been made [35, 40].

## 2. Materials and Methods

### 2.1. Animals and Surgical Methods

All experimental procedures conformed to the Guidelines for the Use of Animals in Research of the Society for Neuroscience and were approved by the Institutional Animal Care and Use Committee of Emory University. All animal surgeries and euthanasia were performed with appropriate general anesthesia in an effort to minimize suffering. Experiments were conducted on adult (>2 months old) mice.

In this study, mouse genetics was exploited to evaluate the role of motoneuronal BDNF in synaptic withdrawal following peripheral nerve injury and any effect of posttransection exercise. Experiments were conducted using 40 female mice, all on a C57BL/6J background. Twenty-eight mice were wild-type mice and 12 were conditional BDNF knockout mice. The BDNF knockout mice were developed using the Cre-lox system with an animal known as single-neuron labeling with inducible Cre-mediated knockout (SLICK) [41]. In these animals, a tamoxifen-inducible Cre and yellow fluorescent protein (YFP) are expressed under the control of the* thy-1* promoter. The tamoxifen-inducible Cre is found in a subset of all motoneurons and dorsal root ganglion (DRG) neurons and is noted by the coupled expression of YFP [34, 41]. Founders of the A strain of SLICK mice (B6.Cg-Tg (Thy1-cre/ESR1,-EYFP)AGfng/J, stock number 007606) were obtained from the Jackson Laboratories (Bar Harbor, ME). These mice were bred with transgenic mice in which loxP sites were inserted flanking the coding region (exon IX) of the BDNF gene (floxed BDNF mice, a generous gift of Dr. Michael Sendtner, [42]) and back-crossed until homozygous for the floxed allele (SLICK::BDNF^f/f^) to create conditional BDNF knockout mice.

Mice were assigned to one of six groups. Numbers of mice used in the different experimental groups are shown in Table 1. The numbers of animals studied in each group were selected based on the results of an* a priori* power sample size estimate [43], using data from our previously published studies [7] and alpha = 0.05 and power = 0.8. A true difference in means that is smaller than the observed difference in mean synaptic coverage between intact and injured mice in that previous study was found for both VGLUT1 and GAD67, when *N* = 3 animals per group. To be on the safe side, we originally selected at least four mice in each treatment group studied, but in the transected SLICK::BDNF^f/f^ & exercised group, one animal died unexpectedly and was not replaced.

In all but the control and intact SLICK::BDNF^f/f^ mice, the right sciatic nerve was cut in the mid-thigh, under isoflurane anesthesia. Because retrogradely transported neurotrophic molecules originating from transformed Schwann cells in the distal nerve segment or even their muscle targets [44, 45] are the same as those postulated to act as retrograde signals from motoneurons that could influence the withdrawal of synapses, a section of the nerve was removed to avoid reinnervation. Alvarez et al. [6] found the same amount of synaptic withdrawal following nerve transection whether the cut nerves were repaired or ligated to prevent reinnervation. However, we wanted to reduce the probability that any retrograde signals promoted by exercise came from sources other than the motoneurons. Therefore, the proximal segment of the cut nerve was left disconnected from the distal segment. Spinal motoneurons in these animals were labeled by application of dextran (MW 10,000) conjugated to a red fluorophore (either Alexafluor 594 or Alexafluor 555, Invitrogen, Eugene, OR, http://www.lifetechnologies.com/) onto the proximal stumps of the cut nerves [46, 47]. In the Control and Intact SLICK::BDNF^f/f^ mice, the sciatic nerves were left intact, and, in these mice, the motoneuron cell bodies were labeled by injection (1 *μ*L) of the cholera toxin B subunit conjugated with the aforementioned Alexafluors (Invitrogen) (1 *μ*g/*μ*L in distilled water) into the gastrocnemius and tibialis anterior muscles.

### 2.2. Tamoxifen Treatment

All mice were genotyped from tail DNA prior to selection for treatment. Tamoxifen was administered orally to adult SLICK::BDNF^f/f^ mice in two rounds of three days per week treatments with two weeks in-between. Dosages were calculated based on the weight of the mouse at 0.75 mg/20 g, and the tamoxifen was dissolved at a concentration of 10 mg/mL in sunflower seed oil. All treated mice were not used until at least two weeks following the last day of tamoxifen treatment. We have shown previously that this brief tamoxifen treatment is effective in knocking out the BDNF gene in SLICK::BDNF^f/f^ mice [34].

### 2.3. Treadmill Exercise Procedures

For nine wild-type mice and three SLICK::BDNF^f/f^ mice, exercise was begun on the third day following nerve repair surgery and was conducted five days per week for two weeks (Table 1). An interval treadmill exercise paradigm was used (20 m/min, 4 × 2 min/5 min rest, 5 days/week) because it resembles the pattern of voluntary running observed in mice when a wheel is placed in their cages [48] and because we have shown that it is effective on synaptic withdrawal following peripheral nerve transection in female mice [7, 35]. We have described this exercise pattern in detail in our previous papers [49, 50].

### 2.4. Immunohistofluorescence Analysis

All injured animals were euthanized two weeks following sciatic nerve transection. Intact SLICK::BDNF^f/f^ mice were euthanized two weeks after the end of their tamoxifen treatment. All mice were deeply anesthetized with pentobarbital (150 mg/kg, i.p.) and transcardially perfused with 50 ml of normal saline followed by 100 ml of periodate-lysate-paraformaldehyde fixative [51]. Spinal cord segments L3–L6 were harvested and cryoprotected in 20% sucrose. Spinal cords were sectioned on a cryostat in the transverse plane at 20 *μ*m.

To visualize the contacts made by structures containing different synapse-associated proteins onto motoneurons, separate sets of tissue sections were incubated with solutions containing one of two different synapse-associated antigens. Glutamate decarboxylase (GAD) is the rate-limiting enzyme in the conversion of L-glutamate into the inhibitory neurotransmitter, gamma-aminobutyric acid (GABA). Two GAD isoforms are known by their molecular weights as GAD67 and GAD65. The majority of GABAergic boutons in the ventral horn of the spinal cord are strongly immunoreactive for GAD67 and originate from interneurons in the spinal cord [52]. Synaptic contacts made by GAD67 immunoreactive structures contacting motoneurons decrease significantly following peripheral nerve transection, but by 12 weeks after such injuries, many of them are restored [6]. The overwhelming majority of terminals in the spinal cord containing the VGLUT1 isoform are from primary afferent neurons [53]. They are withdrawn permanently after transection of peripheral nerves [6]. We evaluated immunoreactivity to these two examples of different kinds of synaptic inputs to motoneurons in all animals in this study.

Sections were first incubated for 1 hour at room temperature in buffer containing 0.1 M phosphate-buffered saline (PBS) with 0.4% Triton X (PBS-T) and 10% normal goat serum (NGS). Following preincubation, the tissues were incubated in primary antibodies, diluted in 1 : 2000 for rabbit anti-VGLUT1 (Synaptic Systems GmbH number TO30326, Göttingen, Germany, http://www.sysy.com/) and 1 : 200 for mouse anti-GAD67 (Millipore number MAB5406, Billerica, MA, USA, http://www.merckmillipore.com/), for 24 hours at room temperature. After washing in 0.1 M PBS for 3 times, the sections were incubated with secondary antibodies (Alexafluor 647, goat anti-rabbit IgG or goat anti-mouse IgG) for 2 hours at room temperature to detect immunofluorescence. All the incubations and reactions were separated by 3 × 10 min washes in 0.1 M PBS.

### 2.5. Imaging Analysis

**Figure 1:**
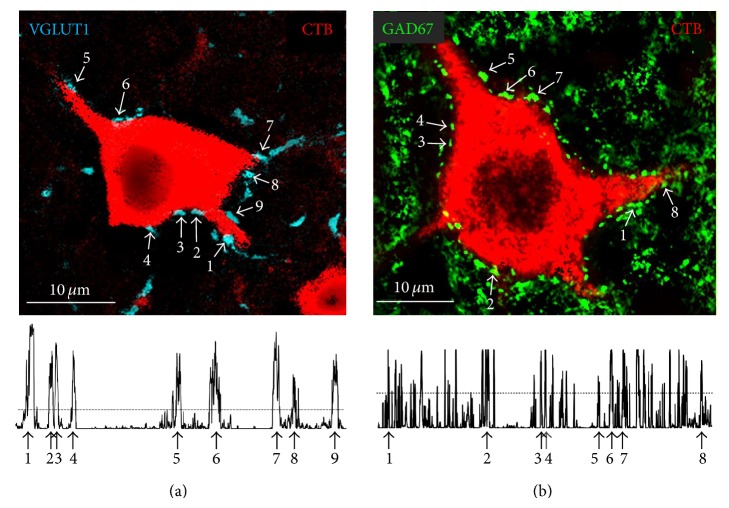
Images containing motoneurons retrogradely labeled with the beta subunit of cholera toxin (CTB) (red) are shown from spinal cord sections from intact wild-type mice to illustrate the ways that measurements of synaptic coverage were made. In panel (a), immunoreactivity to vesicular glutamate transporter 1 (VGLUT1) is marked by cyan. In panel (b), immunoreactivity to glutamic acid decarboxylase (GAD67) is shown in green. Each image is from a single optical section of the cell made through the nucleus. In all of the cells studied, the perimeter of the cell was outlined to create a region of interest (ROI) and a profile plot (shown below each image) of the fluorescence intensity along the ROI, in the appropriate channel of the RGB image, was obtained using Image J. The locations of some, but not all, prominent VGLUT1 or GAD67 immunoreactive terminals in contact with the motoneuron soma are indicated by numbers in the micrographs and the labeled peaks on the associated profile plot. The dotted line across each profile plot represents the threshold used to define the amount of synaptic coverage in this cell. Portions of the profile plot above this line are considered regions of the cell surface that are contacted by structures immunoreactive for these synapse-associated proteins.

Sections were viewed with a laser scanning confocal microscope (LSM-510, Zeiss). The experimenter was blinded to the experimental grouping of the source of tissues on slides while capturing images and collecting data. In all of the spinal cords studied, labeled motoneurons were selected for study throughout the caudorostral extent of the marked motor nucleus to provide a reasonable sample of all of the labeled cells. Cells were selected for study only if they contained retrograde fluorescent tracer that filled the somata and extended into the proximal-most dendrites and their cell borders could be recognized. High magnification (63x) images of individual labeled motoneuron cell bodies were obtained at a confocal Z-step of 1 *μ*m. Optical sections were selected for analysis only if they included a region of the motoneuron soma containing a visible nuclear shadow (Figure 1(a)). In the SLICK::BDNF^f/f^ mice, motoneurons that contained both the retrograde label and YFP and motoneurons containing the retrograde label but no YFP were studied separately. Because we have shown the effectiveness of this cell-type specific knockout of this gene previously [34], we assumed that YFP+ cells in these mice were null for BDNF and those that lacked YFP were equivalent to cells found in wild-type mice.

To measure the extent of contacts made by structures immunoreactive to these synapse-associated antigens on the somata and proximal-most dendrites of the labeled motoneurons, we used ImageJ software as described by Wang et al. [54]. For each motoneuron selected for study, a region of interest (ROI) was created around about the perimeter of the cell profile containing the retrograde fluorescent tracer, using the thresholding feature of ImageJ. When establishing the ROI, only the red channel of the RGB images, containing the retrograde label in the motoneuron, was used. Perhaps because of the relatively thin optical sections used, the ROIs included only proximal-most dendrites extending no longer than 25 *μ*m from the center of the cell somata. The width of this line defining the ROI was then set to 1 *μ*m, and a plot profile (Figure 1) was created by measuring the pixel (fluorescence) intensity beneath this ROI for each motoneuron, in the blue (Alexafluor 647) channel of the RGB image (cyan in Figure 1(a)). For each cell studied, a fluorescence intensity threshold was established as the mean plus the 95% confidence interval of fluorescence intensity measured within the ROI in the blue channel of the RGB image (Figure 1(a), horizontal dotted line). We assumed that any immunofluorescence for synapse-associated proteins inside the cell boundaries would be nonspecific. The proportion of the values in the plot profile that exceeded this threshold was calculated and expressed as a proportion of the cell profile perimeter or the percent synaptic coverage. Mean values of the percentage of synaptic coverage were determined from each of the cells sampled in each mouse. In all wild-type mice, 10–20 motoneurons were studied in each mouse. For the SLICK::BDNF^f/f^ mice, where only ca. 10% of all motoneurons expressed Cre recombinase and YFP [41], a smaller number of YFP+ and YFP− motoneurons were studied in each mouse (Table 1). As above, retrogradely labeled YFP+ and YFP− motoneurons were sampled at caudorostral positions throughout the sciatic motor nucleus.

### 2.6. Statistical Analysis

Mice were assigned to different treatment groups (Table 1). Average synaptic coverage for the different markers used was determined in each mouse, and this mean became a data point in one group. In the knockout mice, mean coverage was determined for YFP+ and YFP− cells from the same animals. Significance of differences in mean synaptic coverage between groups and between data points within groups was evaluated using analysis of variance (ANOVA) (Statistica, StatSoft, Tulsa, OK). When the omnibus test for significance was met (*P* < 0.05), paired post hoc testing (Tukey's honest significant differences (HSD)) was applied.

## 3. Results

### 3.1. Effect of Treadmill Exercise on Synaptic Withdrawal

**Figure 2:**
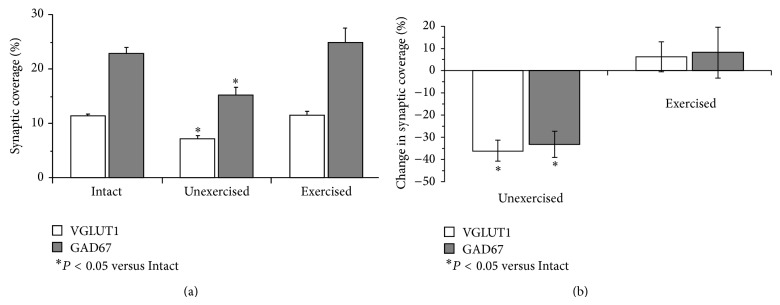
(a) The mean (± SEM) percent coverage by synaptic structures contacting motoneuron somata and proximal-most dendrites is shown for intact wild-type mice and two weeks after transection of the sciatic nerve, in unexercised mice and mice exercised daily, five days per week during that period. Data are shown for synaptic contacts that were immunoreactive for VGLUT1 (white bars) or GAD67 (grey bars). (b) The same data as in (a) are plotted as the mean (±95 confidence interval) percent reduction in synaptic contact, relative to intact mice. *n* = 11 for intact, *n* = 8 for unexercised, and *n* = 9 for exercised.

The extent of withdrawal of synaptic contacts onto motoneurons of wild-type mice two weeks following sciatic nerve transection, with or without exercise, is summarized in Figure 2. The average (±SEM) percent coverage of motoneuron somata and proximal-most dendrites by structures immunoreactive to these VGLUT1 and GAD67 is shown in Figure 2(a). The results of the ANOVA for synaptic coverage by both excitatory synapses originating from primary afferent neurons (VGLUT1) (*F*_2,18_ = 25.55, *P* < 0.01) and for inhibitory synapses originating from interneurons in the spinal cord (GAD67) (*F*_2,28_ = 10.76, *P* < 0.01) were significant. In unexercised mice two weeks following sciatic nerve injury, we found significant reductions in coverage for both markers, relative to coverage in Intact mice (Tukey's HSD, *P* < 0.01). In mice treadmill exercised moderately five days per week during the same survival period, no significant differences in the extent of contacts made by either of these synapse-associated proteins were found, relative to Intact controls (Tukey's HSD, n.s.).

The same data are shown as the mean (±95% confidence limits) percent reduction in synaptic coverage, relative to intact mice, in Figure 2(b). When these data for proportional changes in synaptic coverage were subjected to ANOVA, a significant difference was found (*F*_3,14_ = 3.61, *P* < 0.05). Compared to intact mice, similar significant (HSD, *P* < 0.01) reductions in synaptic coverage (VGLUT1 mean = −36.04%, SEM = 4.49%; GAD67 M = −33.23%, SEM = 5.92%) were found for both VGLUT1+ and GAD67+ synaptic contacts in unexercised mice. No significant changes in synaptic coverage were found in treadmill exercised animals (HSD, n.s.). Thus, moderate exercise applied during the first two weeks following peripheral nerve transection injury has a profound effect on synaptic withdrawal.

### 3.2. Synaptic Withdrawal in BDNF Knockout Mice

We used the Cre-lox system to generate SLICK::BDNF^f/f^ mice in which, once they have been treated with tamoxifen, the BDNF gene is knocked out in YFP-expressing neurons. In the spinal cords of these mice, YFP is expressed mainly in motoneurons, but also in some other neurons. We were concerned that tamoxifen-induced changes in BDNF expression in these nonmotoneuron populations could contribute to changes in the synaptic inputs onto motoneurons that we have studied. Therefore, we measured the coverage of the somata of retrogradely labeled motoneurons by YFP+ synaptic inputs. In a series of four SLICK::BDNF^f/f^ mice not treated with tamoxifen, synaptic coverage by YFP+ structures was very small (mean = 0.31%, SEM = 0.13%). After treatment with tamoxifen, this proportion did not change significantly (M = 0.99%, SEM = 0.37%, unpaired *t*-test, *t*(4) = −1.74, n.s.). Thus, we assume that the contribution to our data set of synapses from neurons in which the BDNF was manipulated is negligible.

**Figure 3:**
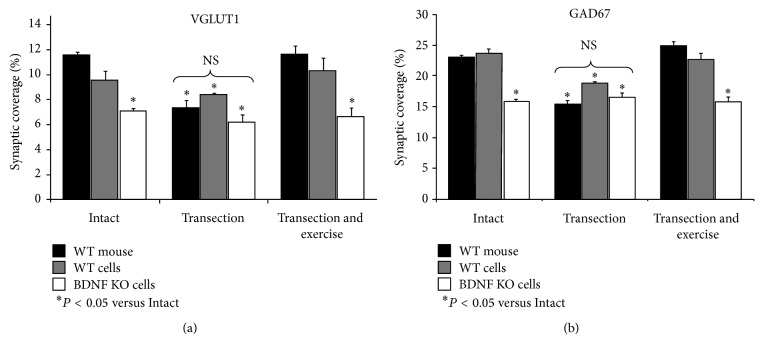
The mean (+SEM) percent of motoneuron somata and proximal dendrites that are contacted by structures immunoreactive for VGLUT1 (a) and GAD67 (b) in WT mice and for WT and KO cells in tamoxifen-treated SLICK::BDNF^f/f^ mice are shown. Separate groups of three bars are shown in each panel for data from intact mice (left), unexercised mice two weeks after sciatic nerve transection (center), and two weeks after nerve transection and five days per week exercise (right) (*n* = 5, 4, and 3 mice, resp.). The left group of results shows that synaptic coverage is significantly reduced in BDNF KO cells compared to WT mouse and WT cells in intact mice. Following nerve transection, synaptic coverage is significantly decreased in all cells, but coverage in BDNF KO cells is not changed from those seen in the intact animal. Exercise following transection restores synaptic coverage in all cells except the BDNF KO cells.

In SLICK::BDNF^f/f^ mice treated with tamoxifen, retrogradely labeled motoneurons that also expressed YFP were considered null for the BDNF gene and are referred to here as BDNF- KO cells [34]. Labeled motoneurons that did not also express YFP are referred to here as wild-type cells. The extent of synaptic coverage in tamoxifen-treated SLICK::BDNF^f/f^ mice is summarized in Figure 3 for VGLUT1+ (a) and GAD67+ (b) structures. In each panel, mean (±SEM) coverage on wild-type motoneurons (Figure 3, grey bars) and BDNF-KO motoneurons (Figure 3, white bars) is shown for intact sciatic nerves (left), from mice two weeks after sciatic nerve transection without exercise (center), and from mice two weeks after sciatic nerve transection with exercise (right). Data from wild-type mice (the same data as shown in Figure 2) are shown for comparison (Figure 3, black bars). Significant differences were found in contacts made by VGLUT1+ (*F*_8,25_ = 7.95, *P* < 0.01) and GAD67+ (*F*_8,25_ = 14.18, *P* < 0.01) structures. In tamoxifen-treated SLICK::BDNF^f/f^ mice with intact sciatic nerves, the small differences in mean synaptic coverage onto wild-type (YFP−) motoneurons and motoneurons from intact wild-type mice were not statistically significantly different (HSD, n.s.). Synaptic coverage onto BDNF KO (YFP+) motoneurons in the spinal cords of the same animals was reduced markedly in comparison to that observed in both YFP− cells of the same animals and intact wild-type mice, for both VGLUT1+ and GAD67+ structures (HSD, *P* < 0.01). The reduction in synaptic coverage onto the BDNF KO motoneurons was assumed to be the result of a postsynaptic BDNF knockout, since no other experimental perturbation was applied to these intact mice.

After sciatic nerve transection without exercise, synaptic coverage was reduced markedly in wild-type animals (as noted above) but also in wild-type cells in tamoxifen-treated SLICK::BDNF^f/f^ mice (HSD, *P* < 0.01). In BDNF KO cells in tamoxifen-treated SLICK::BDNF^f/f^ mice, synaptic coverage was not significantly changed after sciatic nerve transection, relative to that of BDNF KO cells of intact mice of the same genotype. Coverage in axotomized BDNF KO cells in tamoxifen-treated SLICK::BDNF^f/f^ mice also was not significantly different from that in axotomized wild-type cells of the same animals.

In wild-type cells of tamoxifen-treated SLICK::BDNF^f/f^ mice whose sciatic nerve had been cut and who were then exercised daily for two weeks, synaptic coverage was similar to that noted in wild-type cells in intact mice and to wild-type mice that had been exercised following sciatic nerve transection (HSD, n.s.), as described above for wild-type mice. No such effect of exercise was found for BDNF KO cells in tamoxifen-treated SLICK::BDNF^f/f^ mice. The extent of contacts made with motoneurons lacking the BDNF gene by structures immunoreactive for either VGLUT1+ or GAD67+ was significantly reduced in exercised animals but was not different from BDNF KO cells in intact animals.

## 4. Discussion

Following peripheral nerve transection, both sensory and motor targets in the periphery are disconnected from the CNS, and a substantial portion of the synaptic inputs onto the axotomized motoneurons are withdrawn. This synaptic stripping could contribute substantially to the poor functional recovery from peripheral nerve injuries found clinically. If mice are treated with two weeks of daily treadmill exercise following peripheral nerve injury, the anticipated loss of synaptic coverage on the axotomized motoneurons is not noted [7, 35, 36]. We have measured the extent of contacts made by two different types of synaptic inputs to these motoneurons using antibodies to synapse-associated proteins, and we assume that all contacts made by immunoreactive structures represent similar synaptic inputs. These two types of synaptic inputs were studied as models of the effects of exercise on synaptic stripping on two very different types of synapses. Whether or not the contacts established in response to exercise represent synaptic inputs that are equivalent, both anatomically and functionally, to the withdrawn synaptic inputs is not known at this time.

The cellular mechanism that triggers synaptic stripping following peripheral nerve transection remains incompletely understood. In early reports, glial cells were proposed to be involved. The robust proliferation and process extension of microglia surrounding axotomized motoneurons (e.g., [16]) are said to be instrumental in the removal or stripping of synapses [5]. However, if this microglial reaction to peripheral nerve transection is blocked, synaptic stripping still occurs in response to peripheral axotomy (reviewed in [22]). Similarly, the somewhat later influx of reactive astrocytes to the region of the axotomized motoneurons, which may be stimulated by microglial cells [55], is thought to play a role in active removal of synapses after nerve transection [17], but nerve injury-induced effects on astrocytes and microglia are quantitatively similar in different mouse strains in which the extent of synaptic stripping is very different [21, 23].

Synaptic withdrawal also is postulated to result from a loss of molecules from the axotomized motoneurons that normally contribute to synaptic stability [25]. A case has been made that NO contributes in this manner [26]. One aspect of the effect of production of NO by axotomized motoneurons is a decrease in secretion of BDNF [27]. During the first week following peripheral nerve injury, before much synaptic stripping occurs, BDNF mRNA is upregulated transiently, but then it is downregulated rapidly in axotomized motoneurons [28], coinciding with the onset of synaptic withdrawal. After application of recombinant human neurotrophins to the proximal stumps of cut nerves, no evidence for synaptic stripping is found [29, 30, 56]. Based on these observations, it would seem that neurotrophins are* sufficient* to restore or retain synaptic inputs onto axotomized motoneurons. Consistent with this model, when we treated SLICK::BDNF^f/f^ mice with tamoxifen, thereby inducing knockout of the BDNF gene in YFP+ motoneurons, and simulating the decline in BDNF expression found after peripheral axotomy, we observed a rapid withdrawal of contacts made by both VGLUT1+ and GAD67+ structures on those neurons. The extent of that withdrawal was indistinguishable from the amount of withdrawal that occurred following peripheral nerve transection. However, eliminating BDNF production by motoneurons did not result in a complete loss of synaptic inputs; some VGLUT1+ and GAD67+ contacts remain. Neuronal BDNF production may not be* necessary* for the stabilization of* all *synaptic inputs. Some inputs may be sensitive to the loss of BDNF while other inputs are not. It is also possible that the loss of motoneuronal BDNF after peripheral axotomy, or in our knockout mice, may be only a part of the synaptic withdrawal mechanism. Further studies are needed to more precisely evaluate the relationship between BDNF content in the postsynaptic motoneurons and their synaptic coverage.

Because treadmill exercise is known to increase the expression of neurotrophins, such as BDNF in spinal motoneurons [31], and also to have a marked effect on synaptic stripping [7, 35, 36], we hypothesized that the effect of treadmill exercise on synaptic stripping is the result of an increase in the expression of BDNF in the axotomized motoneurons. Our hypothesis was tested using mice in which the gene for BDNF was knocked out in a subset of motoneurons in the lumbar spinal cord only after treatment with tamoxifen [41]. Exercise influenced synaptic coverage onto the wild-type motoneurons but not the knockout cells in the very same animals. These findings are consistent with our hypothesis. We believe that they are strong evidence that motoneuronal BDNF is* necessary* for the effects of exercise on synaptic stripping following peripheral nerve injury.

The simplest model for this role of BDNF is that it is secreted by the postsynaptic motoneurons and acts as a retrograde signal by binding to trkB molecules on synaptic inputs. Through the action of downstream effectors of the trkB signaling pathway, the cytoskeleton of the synaptic terminals might be stabilized. Increasing BDNF expression in motoneurons using exercise, as in the present study, or the application of retrogradely transported neurotrophins [29, 30, 56] might be expected to reverse or prevent these cellular events in wild-type motoneurons and result in the appearance of axotomized motoneurons with full compliments of synaptic inputs, as has been observed. Consistent with this simplest of models, at least some synaptic terminals onto motoneurons have been shown to express the full length trkB protein [57]. However, the postsynaptic motoneurons also express the full length trkB receptor [58], and glial cells are known to contain both full length and truncated trkB receptors [59–61]. Evidence for signaling between motoneurons and their synaptic inputs through a more complex autocrine/paracrine mechanism has been acquired in preliminary experiments with motoneuron-specific trkB knockout mice [62]. Such a mechanism cannot be ruled out as an explanation of the effect of exercise on synaptic inputs to motoneurons after peripheral nerve injury at this time. Future studies should examine synaptic withdrawal after manipulating these different populations of trkB receptors.

Our finding that manipulation of motoneuronal BDNF expression resulted in withdrawal of inhibitory synapses is consistent with the results of previous studies. For example, BDNF has been implicated in the formation of inhibitory synapses in other brain regions [63–65]. Recombinant human BDNF treatments following peripheral injury are linked to the formation of inhibitory synapses [66]. The finding that BDNF depletion from motoneurons also resulted in the withdrawal of VGLUT1+ excitatory synapses was surprising, as these synapses have been associated with NT-3. Administration of recombinant NT-3 reduced (excitatory and inhibitory) synaptic withdrawal from axotomized motoneurons [29], resulted in a large increase in the amplitude of monosynaptic EPSPs in axotomized spinal motoneurons [30], and restored connections of group Ia afferent neurons onto motoneurons in knockout mice that do not develop muscle spindles [67]. Like BDNF, NT-3 expression in motoneurons is stimulated by treadmill exercise [32], albeit at a somewhat later time than BDNF. We have postulated that BDNF, produced by motoneurons, is a part of a cellular mechanism that retains both VGLUT1+ and GAD67+ synapses on spinal motoneurons. It is possible that NT-3 might act in concert with BDNF, as has been suggested by others [68], perhaps as a downstream target of BDNF acting through trkB receptors. Clearly, more detailed characterization of the roles of BDNF and NT-3 in maintenance of VGLTUT1+ synapses onto motoneurons is required.

Synaptic inputs from different sources are withdrawn from motoneurons following peripheral nerve injury, and most are restored later, whether or not axon regeneration in the periphery is successful. However, synapses from group Ia afferent neurons, which are immunoreactive for VGLUT1, are not restored and are withdrawn permanently [6]. Thus we suggest that reduction of motoneuronal BDNF expression might contribute to the original withdrawal of all types of synapses immediately following peripheral nerve injury but, as suggested by others [6, 69], other factors, such as injury to the peripheral processes of axons of dorsal root ganglion neurons, contribute to the unique permanent loss of VGLUT1+ inputs.

We show here and elsewhere [7, 70] that moderate exercise in the form of treadmill walking has a positive effect on synaptic plasticity in the spinal cord following peripheral nerve injury. In exercised animals, there is no evidence of synaptic withdrawal after sciatic nerve transection and repair (unlike the profound withdrawal found in untreated mice). An important question that arises is whether the effect of exercise maintains the existing synapses or simply delays the onset of axotomy-induced synaptic withdrawal. In a recent paper using rats, we [71] demonstrated that if animals are exercised beginning on the 3rd day after nerve transection, coverage by VGLUT1+ and GAD67+ contacts 10 weeks after transection is not significantly different from the coverage in intact animals. In contrast, exercise begun 3 weeks after transection is not effective in maintaining or restoring VGLUT1+ contacts. Thus, the short period of exercise has a long-lasting effect if applied immediately after injury in rats. Whether the same is true in mice is not known at this time.

Synaptic withdrawal following peripheral nerve injury reduces the overall responsiveness of the axotomized motoneurons to afferent inputs, either from the periphery or from circuits within the CNS, and might be considered a potent contributor to the poor functional recovery found following peripheral nerve injuries [1, 2]. The complete loss of the stretch reflex from reinnervated muscles [13, 14] that is related to a permanent loss of afferent terminals on motoneurons [6, 72] is a striking example of such a functional deficit. In this context, the effect of exercise on synaptic inputs might be seen as a means by which functional recovery following peripheral nerve injury could be enhanced.

## 5. Conclusions

Moderate exercise applied following peripheral nerve injury results in retention or restoration of synaptic inputs onto the axotomized motoneurons that is dependent on the synthesis and secretion of brain-derived neurotrophic factor (BDNF) by the postsynaptic neurons. BDNF produced by spinal motoneurons is a part of a cellular mechanism that retains synaptic inputs onto those cells.

## Figures and Tables

**Table 1:** Groups and numbers of mice studied.

Group	Number of mice	Number of cells/mouse
Intact^*^	11	10–20
Transected & unexercised^*^	8	20
Transected & treadmill exercised^*^	9	20
Intact SLICK::BDNF^f/f^	5	12^†^
Transected SLICK::BDNF^f/f^ & unexercised	4	12^†^
Transected SLICK::BDNF^f/f^ & exercised	3	12^†^

^*^WT mice.

^†^Six WT and six KO cells were studied in each SLICK::BDNF^f/f^ KO mouse. All knockout mice were tamoxifen-treated.
